# Supplementary material for: From crisis to responsibility: The role of industry type, leadership style, and regulatory environment in shaping post-COVID-19 CSR initiative
Source: PLoS One. 2024 Apr 18;19(4):e0292732. doi: 10.1371/journal.pone.0292732 (PMC11025823; doi:10.1371/journal.pone.0292732)
Supplement: Attachment — Submitted filename: PONE-D-23-20140.docx [file pone.0292732.s002.docx]

PLOS ONE

**From Crisis to Responsibility: The Role of Industry Type, Leadership Style, and Regulatory Environment in Shaping Post-COVID-19 CSR Initiative.**

--Manuscript Draft--

| **Manuscript Number:** | PONE-D-23-20140 |
| --- | --- |
| **Article Type:** | Research Article |
| **Full Title:** | From Crisis to Responsibility: The Role of Industry Type, Leadership Style, and Regulatory Environment in Shaping Post-COVID-19 CSR Initiative. |
| **Short Title:** | From Crisis to Responsibility |
| **Corresponding Author:** | Saima Kiran Zhengzhou University Zhengzhou, CHINA |
| **Keywords:** | corporate social responsibility; industry type; leadership style; regulatory environment; Environmental protection |
| **Abstract:** | Corporate social responsibility (CSR) is a major concern in modern industries. Chinese industries are growing rapidly and delivering products and services to the market. The Covid-19 pandemic has changed the working style of every type of industry. The objective of this research was to determine the influence of leadership style and industry type on the regulatory environment. This research also aims to determine the impact of the regulatory environment on CSR from the perspective of Chinese industries. Data based on a sample size of 599 was used for data analysis, and Smart PLS 3.0 was used for the results of measurement model assessment and structural model assessment. This study highlighted that industry type and leadership style have a significant positive impact on the regulatory environment and CSR. The framework of this research is based on the identified research gap, and the findings of this study are a significant for Chinese policy makers. Furthermore, the research also asserted practical implications that are reliable to advance practices for the regulatory environment and achieve CSR by Chinese firms. This study has several limitations that are required to be significantly addressed for sustainability of organizations |
| **Order of Authors:** | Yongming Zhu |
|  | Saima Kiran |
|  | Muhammad Salman |
| **Additional Information:** |  |
| **Question** | **Response** |
| **Financial Disclosure**  Enter a financial disclosure statement that describes the sources of funding for the work included in this submission. Review the [submission guidelines](http://journals.plos.org/plosone/s/submission-guidelines#loc-financial-disclosure-statement) for detailed requirements. View published research articles from [*PLOS ONE*](http://journals.plos.org/plosone/) for specific examples.  This statement is required for submission and **will appear in the published article** if the submission is accepted. Please make sure it is accurate. | The authors received no specific funding for this work. |

*Powered by Editorial Manager® and ProduXion Manager® from Aries Systems Corporation*

| **Unfunded studies**  Enter: *The author(s) received no specific funding for this work.*  **Funded studies**  Enter a statement with the following details:   - Initials of the authors who received each award - Grant numbers awarded to each author - The full name of each funder - URL of each funder website - Did the sponsors or funders play any role in the study design, data collection and analysis, decision to publish, or preparation of the manuscript? - **NO** - Include this sentence at the end of your statement: *The funders had no role in study design, data collection and analysis, decision to publish, or preparation of the manuscript.* - **YES** - Specify the role(s) played. |  |
| --- | --- |
| * typeset |  |
| **Competing Interests**  Use the instructions below to enter a competing interest statement for this submission. On behalf of all authors, disclose any [competing interests](http://journals.plos.org/plosone/s/competing-interests) that could be perceived to bias this work—acknowledging all financial support and any other relevant financial or non- financial competing interests.  This statement is required for submission and **will appear in the published article** if the submission is accepted. Please make sure it is accurate and that any funding sources listed in your Funding Information later in the submission form are also declared in your Financial Disclosure statement.  View published research articles from  [*PLOS ONE*](http://journals.plos.org/plosone/) for specific examples. | The authors have declared that no competing interests exist. |

*Powered by Editorial Manager® and ProduXion Manager® from Aries Systems Corporation*

| **NO authors have competing interests**  Enter: *The authors have declared that no competing interests exist.*  **Authors with competing interests**  Enter competing interest details beginning with this statement:  *I have read the journal's policy and the authors of this manuscript have the following competing interests: [insert competing interests here]* |  |
| --- | --- |
| * typeset |  |
| **Ethics Statement**  Enter an ethics statement for this submission. This statement is required if the study involved:   - Human participants - Human specimens or tissue - Vertebrate animals or cephalopods - Vertebrate embryos or tissues - Field research   Write "N/A" if the submission does not require an ethics statement.  General guidance is provided below. Consult the [submission guidelines](http://journals.plos.org/plosone/s/submission-guidelines#loc-guidelines-for-specific-study-types) for detailed instructions. **Make sure that all information entered here is included in the Methods section of the manuscript.** | This study adheres to the guidelines of the ethical review process of the associated university and approved by the ethics committees of the School of Management Science and Engineering, Zhengzhou University, Henan, China. |

*Powered by Editorial Manager® and ProduXion Manager® from Aries Systems Corporation*

| **Format for specific study types**  **Human Subject Research (involving human participants and/or tissue)**   - Give the name of the institutional review board or ethics committee that approved the study - Include the approval number and/or a statement indicating approval of this research - Indicate the form of consent obtained (written/oral) or the reason that consent was not obtained (e.g. the data were analyzed anonymously)   **Animal Research (involving vertebrate animals, embryos or tissues)**   - Provide the name of the Institutional Animal Care and Use Committee (IACUC) or other relevant ethics board that reviewed the study protocol, and indicate whether they approved this research or granted a formal waiver of ethical approval - Include an approval number if one was obtained - If the study involved *non-human primates*, add *additional details* about animal welfare and steps taken to ameliorate suffering - If anesthesia, euthanasia, or any kind of animal sacrifice is part of the study, include briefly which substances and/or methods were applied   **Field Research**  Include the following details if this study involves the collection of plant, animal, or other materials from a natural setting:   - Field permit number - Name of the institution or relevant body that granted permission |  |
| --- | --- |
|  |  |
| **Data Availability**  Authors are required to make all data underlying the findings described fully available, without restriction, and from the time of publication. PLOS allows rare exceptions to address legal and ethical concerns. See the [PLOS Data Policy](http://journals.plos.org/plosone/s/data-availability) and [FAQ](http://journals.plos.org/plosone/s/data-availability#loc-faqs-for-data-policy) for detailed information. | Yes - all data are fully available without restriction |

*Powered by Editorial Manager® and ProduXion Manager® from Aries Systems Corporation*

| A Data Availability Statement describing where the data can be found is required at submission. Your answers to this question constitute the Data Availability Statement and **will be published in the article**, if accepted. |  |
| --- | --- |
| **Important:** Stating ‘data available on request from the author’ is not sufficient. If your data are only available upon request, select ‘No’ for the first question and explain your exceptional situation in the text box. |  |
| Do the authors confirm that all data underlying the findings described in their manuscript are fully available without restriction? |  |
| **Describe where the data may be found in full sentences. If you are copying our sample text, replace any instances of XXX with the appropriate details.**   - If the data are **held or will be held in a public repository**, include URLs, accession numbers or DOIs. If this information will only be available after acceptance, indicate this by ticking the box below. For example: *All XXX files are available from the XXX database (accession number(s) XXX, XXX.*). - If the data are all contained **within the manuscript and/or Supporting Information files**, enter the following: *All relevant data are within the manuscript and its Supporting Information files.* - If neither of these applies but you are able to provide **details of access elsewhere**, with or without limitations, please do so. For example:   *Data cannot be shared publicly because of [XXX]. Data are available from the XXX Institutional Data Access / Ethics Committee (contact via XXX) for researchers who meet the criteria for access to confidential data.*  *The data underlying the results presented in the study are available from (include the name of the third party* | Primery data has been used |

*Powered by Editorial Manager® and ProduXion Manager® from Aries Systems Corporation*

| *and contact information or URL).*   - This text is appropriate if the data are owned by a third party and authors do not have permission to share the data.   * typeset |  |
| --- | --- |
| Additional data availability information: |  |

*Powered by Editorial Manager® and ProduXion Manager® from Aries Systems Corporation*

Manuscript	[Click here to access/download;Manuscript;MANUSCRIPT CSR WITH AUTHOR DETAIL.docx](https://www.editorialmanager.com/pone/download.aspx?id=33857604&guid=826a12f8-5cab-42f5-8cdb-baa30405d5ca&scheme=1)

# From Crisis to Responsibility: The Role of Industry Type, Leadership Style, and Regulatory Environment in Shaping Post-COVID-19 CSR Initiative

Yongming Zhu^1^, Saima Kiran^1^, and Muhammad Salman^1^,

1. School of Management Science and Engineering, Zhengzhou University, Henan, China [Zhuyongming@zzu.edu.cn](mailto:Zhuyongming@zzu.edu.cn)

[Saim1234779@gmail.com](mailto:Saim1234779@gmail.com)

[Salman.muhammad2017@yahoo.com](mailto:Salman.muhammad2017@yahoo.com)

Corresponding Author: Saima Kiran ([saim1234779@gmail.com](mailto:saim1234779@gmail.com))

# Abstract

Corporate social responsibility (CSR) is a major concern in modern industries. Chinese industries are growing rapidly and delivering products and services to the market. The Covid-19 pandemic has changed the working style of every type of industry. The objective of this research was to determine the influence of leadership style and industry type on the regulatory environment. This research also aims to determine the impact of the regulatory environment on CSR from the perspective of Chinese industries. Data based on a sample size of 599 was used for data analysis, and Smart PLS 3.0 was used for the results of measurement model assessment and structural model assessment. This study highlighted that industry type and leadership style have a significant positive impact on the regulatory environment and CSR. The framework of this research is based on the identified research gap, and the findings of this study are a significant for Chinese policy makers. Furthermore, the research also asserted practical implications that are reliable to advance practices for the regulatory environment and achieve CSR by Chinese firms. This study has several limitations that are required to be significantly addressed for sustainability of organizations.

**Keywords.** Corporate social responsibility, industry type, leadership style, regulatory environment, environmental protection

# 1. Introduction

In this era of rapid industrialization, social goals are required to be achieved by different kinds of industries. The responsibility of industrial organizations either in manufacturing or in the service sector is required to improve the practices for organizational sustainability (D. Zhang, Lu, Morse, & Liu, 2022). Sustainable development is required to be improved for the safety of the environment. The stability of the environment is necessary for modern organizations that are required to be improved over time (Teng, Teng, Wu, & Chang, 2021). The access of industry to environmental regulations can be a helpful strategy to increase organizational performance. The concern of the industries should be to develop strategies that would be effective to work for the upgradation of the environment (Freze et al., 2023). When there is no sustainability in the environment, the industrial sector has to work on it. After the outbreak of the Covid-19 pandemic, the situation changed altogether regarding industrial performance, and less attention was paid to the goals of corporations regarding the sustainability of the environment.

Corporate social responsibility (CSR) is considered one of the important factors that are influencing organizational work and performance. Organizations that consider themselves responsible to achieve their organizational goals, these organizations are fairly working to achieve sustainability (B. Li, Zhong, Zhang, & Hua, 2021). Meanwhile, the functioning of any organization in environmental terms would be a way forward to achieve sustainability. China is the largest

country from the perspective of primary, secondary, and tertiary industries (Hsiao, Huang, Kang, & Li, 2022). The working of this industrial sector required advanced working to improve organizational performance. The corporate level working in any country requires extra responsibilities that should be managed appropriately for the people (Sadiq et al., 2022). The reliability of organizational working for environmental concerns can be a way forward to get stability in the environment. Industrial work after covid-19 changed the way of traditional practices which could be a negative way to advance organizational performance.

Hao, Xiao, and Chon (2020) highlighted that it is the responsibility of the manufacturing industry management to protect the environment. The manufacturing units are required to follow the strict guidelines that are necessary for the stability of the environment. Furthermore, Hsiao, Jiang, Lian, and Wang (2022) reported that the advanced working of the organization sector and improved performance of employees with efficiency can be a way forward to achieve sustainability in the environment. Many firms are working to advance their performance for achieving organizational sustainability which is necessary for the productive performance of organizations (Y. Zhang, Li, Lei, & Yan, 2023). The firm performance for environmental sustainability depends on different factors, and organizational sustainability is also one of these factors. The access to information shared for public benefit should be improved, and people must be provided with the opportunity to increase their organizational behavior. Broadstock, Chan, Cheng, and Wang (2021) highlighted that the environmental policies should be implemented strictly for those organizations that are contributing negatively to the environmental improvements.

Based on these studies, the literature doesn’t explain the relationship of CSR with industry type and leadership style. In this way, the objective of this research is to determine the influence of leadership style and industry type on the regulatory environment. Furthermore, this research also aims to determine the impact of the regulatory environment on CSR from the perspective of Chinese industries. The framework of this research is based on the research gap, and the novel findings of this study are significant addictions to knowledge. This research has presented remarkable theoretical implications in the body of knowledge. Furthermore, the research also asserted practical implications that are reliable to advance the practices for the regulatory environment and achieving CSR by Chinese industries. This study has several limitations that are required to be addressed significantly for future directions to improve the body of knowledge.

# 2. Review of Literature

2.1 **Underpinning Theory**

The stakeholder theory is a significant theory that discussed the relationships between the employees, firms, and the communities. According to this theory, the stakeholders’ concerns should be focused by the firms (Friedman & Miles, 2006). This theory highlighted that when the stakeholders’ concerns should be considered by the firms, better relationships should be developed (Freeman, Harrison, Wicks, Parmar, & De Colle, 2010). This theory demonstrates that the stakeholders are concerned with a special kind of responsibility that is necessary to improve business practices. Indeed, environmental protection is also the responsibility of businesses, and better opportunities should be developed for the people for the protection of the environment. Based on the conceptual determination of this theory, the research model of the current study is also underpinned by this theory. This study has considered industry type and leadership style as the influencing components that are necessary for the regulatory environment. In this way, this research has considered the relationship between variables based on the theoretical underpinning

of this research. Furthermore, this study has conceptually linked the stakeholder’s theory’s underpinned relationships with CSR. In this way, the framework of this research is developed (see Figure 1), highlighting industry type and leadership style as an independent variable, CSR as the dependent variable, and regulatory environment as a mediating variable.

Leadership Style

Regulatory Environment

Industry Type

CSR

Figure 1. Theoretical Model of Research

# 2.2 Hypotheses Development

The responsibility of every industry is to protect the environment. When any industry is working to improve its practices, the sustainability of the environment is necessary (Freze et al., 2023). The regulation of the environment is necessary for environmental sustainability in the modern time. Furthermore, the success of environmental sustainability is possible when the management of any organization is devoted to working for it (B. Li et al., 2021). Furthermore, the regulation of the government for environmental safety is also appropriate to ensure the working of organizations for the advancement of the environment. Reasonable actions for environmental sustainability are required to be taken over time for its better benefit and advanced working (Hsiao, Jiang, et al., 2022). The reliability of environmental concerns is possible when the management of any organization is devotedly working to improve the environment. The success rate of organizational performance and its advancement can be a way forward to improve environmental sustainability (Broadstock et al., 2021). The awareness of the environment can improve the employees’ working behavior for better achievement of environmental goals by any industry. Industrial goals are achieved when environmental sustainability is to be achieved by the working of any organization (Hu, Qu, Huang, Xue, & Yu, 2022). Modern firms are largely concerned to improve environmental issues by working on realistic strategies to advance organizational performance.

Hypothesis 1: Industry type has a positive impact on the regulatory environment.

The role of leadership is critical to advance organizational performance. The awareness of employees and leadership regarding environmental goals is the way forward to achieve the environmental goals (Sadiq et al., 2022). The safety of the environment for any firm is possible when the people are highly motivated to work for it. The top management strategies developed for the achievement of organizational goals in the best way are necessary to improve organizational performance better for organizational achievement (Hao et al., 2020). Furthermore, those organizations that are not working appropriately to achieve the organizational goals, these organizations are required to improve their performance strategically. The actual working of any organization to advance environmental practices is possible when reasonable actions are taken by the management (M. Zhong, Zhao, & Shahab, 2022). The role of management is critical in organizational sustainability, and the achievement of its goals. When the management of any

organization has clear goals of environmental sustainability, it becomes easy for the employees to follow the management to achieve organizational performance (Waheed et al., 2022). The success rate of organizational work is possible when organizational achievement. The reliability of organizational working and its advanced work for the achievement of organizational goals can be a better strategy to get the advancement of the environment. Many leaders are influencers of the employees to motivate them for better organizational performance.

Hypothesis 2: Leadership style has a positive impact on the regulatory environment.

CSR is necessary for any organization to achieve organizational sustainability and advancement in the environment (Huang, Chen, & Nguyen, 2020). Environmental concerns are achieved when the goals of any organization are realistic to achieve sustainability. A better working approach to organizational performance for environmental upgradation is necessary to advance the environment (Dongfang, Ponce, Yu, Ponce, & Tanveer, 2022). The reliability of environmental concerns and awareness of management for organizational goals is the way forward to achieve sustainability in it. However, when the organizational goals are for advancing the environment, the performance of management and organizational practices are improved to achieve these goals (Poursoleyman, Mansourfar, Hassan, & Homayoun, 2023). The success rate of industry performance for the implementation of CSR is directly dependent on organizational goals. When the goals of any organization are in the right direction, the awareness of organizational sustainability is achieved (L. Zhong, Sun, Law, Li, & Yang, 2022). The improvement in the performance of management is key to achieving the organizational goals. The success rate of the organization working for environmental awareness among the employees can be a better strategy to achieve organizational sustainability. Modern industries are required to work in the environmental sustainability direction to achieve the goals of sustainability (Xu, Chen, Zhou, Dong, & He, 2023). Furthermore, CSR goals are a positive way to achieve sustainability in the environment.

Hypothesis 3: Regulatory environment has a positive impact on corporate social responsibility.

Organizational performance is necessary for the direction of environmental sustainability (Teng et al., 2021). The regulation to protect the environment by any government is necessary to be implemented. The reliability of government regulations for organizational performance is increased over time. The implementation of government regulations for achievement of the sustainability goals is required to be managed by organizational performance (Jaiyeoba, Hossain, Mohd Salleh, & Elgharbawy, 2023). The approach of any industry to work for the sustainability of the environment is necessary. Many organizations are working well to advance their practices for organizational sustainability. The role of every type of industry is to work on the policies that are necessary for organizational sustainability (H. Li, Hu, Zhao, & Li, 2022). The improvement in organizational goals and the worth of organizational working for environmental protection can motivate the employees to work in the right direction for the achievement of the goals of sustainability. However, many organizations are working fairly to achieve the goals of sustainability, and these organizations must protect the environment (Wei, Ayub, & Dagar, 2022). The stable working of top management and long-term policies for organizational sustainability is the way forward to get a better way of organizational advancement. The reliable performance of employees and management of any industry could be a way forward to achieve the goals of CSR.

Hypothesis 4: Regulatory environment has positive mediation between industry type and corporate social responsibility.

The working style of leadership in any industry can influence organizational performance positively and negatively (D. Zhang et al., 2022). Those organizations that have developed appropriate policies to achieve sustainability, these organizations are required to work in a better direction to achieve organizational goals. The reliability of the organizational performance is improved or decreased over time. The organizational management should develop appropriate policies for the safety of the environment, and these policies should be implemented to get a better direction in organizational goals (Hsiao, Huang, et al., 2022). The awareness of management for the achievement of organizational goals is the way forward to get success in organizational performance. The stable working of an organization for the sustainability of the environment is the way forward to achieving organizational goals for environmental sustainability (Y. Zhang et al., 2023). The regulations of government used for the advancement of organizational working can become better strategies to improve organizational performance productively. The better way to improve organizational work can be a factor to advance the sustainability of the environment (Sun & Li, 2021). Many firms are working in the right direction to achieve the organizational goals of sustainability, and the performance of these organizations matters a lot in environmental upgradation.

Hypothesis 5: Regulatory environment has positive mediation between leadership style and corporate social responsibility.

# 3. Methodology

This research has considered reflective measurement items to collect the data. Therefore, a comprehensive process of measurement items development is used in this research. Based on this process, the literature was reviewed at the first stage. Secondly, the operationalization of the study’s variables was made. These variables were considered appropriate to determine its findings. A pool of scale items was created with the help of experts, and the face validity of the measurement items was determined. These scale items were developed based on each dimension of the variables to be measured. However, to determine the validity and reliability of the items, the data was collected from a few respondents of the research population. The population of this research was based on the management of the Chinese industry. Therefore, data from only 80 respondents were collected for the preliminary tests of determination of research items’ reliability and validity. The data was collected after introducing the objective of the research to the respondents. They were asked to provide appropriate data because the analysis would be used for the reliability and validity test of the questionnaire. This collected data was analyzed with AMOS 26 and the findings of exploratory factor analysis and confirmatory factor analysis were determined. The results of exploratory factor analysis reported that the research data has reliability and validity, but the insignificant measurement items with low levels of validity were removed at this stage. Furthermore, the findings of confirmatory factor analysis reported that an appropriate correlation is achieved for research data between the variables. Thus, the developed items for this research are considered appropriate for the final data collection.

The population of this research was the management level employees of the Chinese financial service industry, and the respondents from banks and insurance companies were targeted to collect data. To collect the data convenience sampling approach was used. This approach is best when the population is large and unknown (Taherdoost, 2016). Similarly various recent studies used this approach for data collection (Hua et al., 2023; Tang et al., 2023). A total of 650 questionnaires were surveyed during the month of May 2023 and a brief introduction to the research was also

given to the respondents and the informed verbal consent was taken. The respondents were asked to provide appropriate data for this research. All of their quarries were addressed on time, and 599 questionnaires were collected back. This research has used Smart PLS 3.0 for final data analysis and findings. The results of the measurement model and structural model are used to determine the validity & reliability of the hypotheses and the path findings in this research. These findings are significantly used to determine the final relationships based on theoretical hypotheses with empirical support.

# 4. Data Analysis and Findings

The normality of this research data is tested at the initial stage. The normality test is used to determine the findings of any research to be considered for further analysis. It is necessary to determine the normality of data before utilizing the collected data for final analysis. The findings of missing values are determined at the initial stage to determine the normality. When there are no missing values in the data, then the possibility of data normality increases. Furthermore, the skewness and kurtosis values are considered to test the normality of research data. These values are tested by different methods, but this research has tested skewness and kurtosis findings by inserting the collected data into Smart PLS 3.0. The findings of kurtosis and skewness between -1 and +1 are significantly accepted for data normality (Royston, 1992). These findings are reliable to consider, and the results of this research highlighted that the study has achieved significant validity. The data for skewness, kurtosis, and missing values are reported in Table 1.

Table 1. Data Normality

| No. | Items | Missing | Mean | Median | Min | Max | Standard  Deviation | Excess  Kurtosis | Skewness |
| --- | --- | --- | --- | --- | --- | --- | --- | --- | --- |
| 1 | IT1 | 0 | 4.086 | 4 | 1 | 5 | 1.052 | 0.874 | -0.171 |
| 2 | IT2 | 0 | 3.579 | 4 | 1 | 5 | 1.134 | -0.256 | -0.580 |
| 3 | IT3 | 0 | 3.968 | 4 | 1 | 5 | 1.070 | 0.107 | -0.867 |
| 4 | IT4 | 0 | 3.932 | 4 | 1 | 5 | 1.038 | 0.152 | -0.827 |
| 5 | IT5 | 0 | 3.446 | 4 | 1 | 5 | 1.148 | -0.467 | -0.423 |
| 6 | IT6 | 0 | 3.586 | 4 | 1 | 5 | 1.134 | -0.479 | -0.495 |
| 7 | IT7 | 0 | 3.571 | 4 | 1 | 5 | 1.217 | -0.585 | -0.544 |
| 8 | LS1 | 0 | 4.432 | 5 | 1 | 5 | 0.976 | 0.803 | -0.836 |
| 9 | LS2 | 0 | 4.193 | 5 | 1 | 5 | 1.072 | 0.902 | -0.284 |
| 10 | LS3 | 0 | 3.968 | 4 | 1 | 5 | 1.196 | 0.325 | -0.096 |
| 11 | LS4 | 0 | 4.054 | 4 | 1 | 5 | 1.190 | 0.463 | -0.179 |
| 12 | LS5 | 0 | 3.904 | 4 | 1 | 5 | 1.169 | -0.010 | -0.918 |
| 13 | LS6 | 0 | 4.043 | 4 | 1 | 5 | 1.149 | 0.459 | -0.136 |
| 14 | RE1 | 0 | 4.104 | 4 | 1 | 5 | 1.099 | 0.875 | -0.246 |
| 15 | RE2 | 0 | 3.743 | 4 | 1 | 5 | 1.289 | -0.513 | -0.747 |
| 16 | RE3 | 0 | 3.654 | 4 | 1 | 5 | 1.212 | -0.505 | -0.629 |
| 17 | RE4 | 0 | 3.975 | 4 | 1 | 5 | 1.057 | 0.237 | -0.899 |
| 18 | RE5 | 0 | 4.032 | 4 | 1 | 5 | 1.015 | 0.454 | -0.970 |
| 19 | RE6 | 0 | 3.996 | 4 | 1 | 5 | 1.047 | 0.568 | -0.006 |
| 20 | RE7 | 0 | 4.029 | 4 | 1 | 5 | 1.069 | 0.546 | -0.063 |
| 21 | CSR1 | 0 | 3.943 | 4 | 1 | 5 | 1.136 | 0.296 | -0.991 |

| 22 | CSR2 | 0 | 3.764 | 4 | 1 | 5 | 1.138 | -0.062 | -0.785 |
| --- | --- | --- | --- | --- | --- | --- | --- | --- | --- |
| 23 | CSR3 | 0 | 3.846 | 4 | 1 | 5 | 1.141 | 0.125 | -0.900 |
| 24 | CSR4 | 0 | 4.021 | 4 | 1 | 5 | 1.183 | 0.496 | -0.161 |
| 25 | CSR5 | 0 | 3.714 | 4 | 1 | 5 | 1.104 | -0.220 | -0.633 |

Furthermore, this research has used data analysis by the PLS Algorithm method to determine the reliability and validity of the measurement items at the individual level. For this purpose, the factor loadings are determined. Indeed, the factor loadings are used to determine whether the measurement items used for any variable are reliable or insignificant. The recommended threshold for factor loadings > 0.60 is achieved by the findings of this research (Shevlin & Miles, 1998). In case, if the recommended values are not achieved, the scale items are deleted and data is analyzed again. However, the significant factor loadings values for this research are reported in Table 2. The findings are also displayed in Figure 2.

Factor Loadings

1

0.9

0.8

0.7

0.6

0.5

0.4

0.3

0.2

0.1

0

CSR	Industry Type	Leadership Style	Regulatory Environment

Figure 2. Factor Loadings

CSR1 CSR2 CSR3 CSR4 CSR5 IT1 IT2 IT3 IT4 IT5 IT6 IT7 LS1 LS2 LS3 LS4 LS5 LS6 RE1 RE2 RE3 RE4 RE5 RE6 RE7

Table 2. Factor Loadings

| Items | CSR | Industry Type | Leadership Style | Regulatory  Environment |
| --- | --- | --- | --- | --- |
| CSR1 | 0.904 |  |  |  |
| CSR2 | 0.881 |  |  |  |
| CSR3 | 0.916 |  |  |  |
| CSR4 | 0.788 |  |  |  |
| CSR5 | 0.674 |  |  |  |
| IT1 |  | 0.637 |  |  |
| IT2 |  | 0.684 |  |  |
| IT3 |  | 0.802 |  |  |
| IT4 |  | 0.821 |  |  |
| IT5 |  | 0.778 |  |  |

| IT6 |  | 0.730 |  |  |
| --- | --- | --- | --- | --- |
| IT7 |  | 0.831 |  |  |
| LS1 |  |  | 0.656 |  |
| LS2 |  |  | 0.875 |  |
| LS3 |  |  | 0.849 |  |
| LS4 |  |  | 0.874 |  |
| LS5 |  |  | 0.853 |  |
| LS6 |  |  | 0.849 |  |
| RE1 |  |  |  | 0.630 |
| RE2 |  |  |  | 0.731 |
| RE3 |  |  |  | 0.806 |
| RE4 |  |  |  | 0.836 |
| RE5 |  |  |  | 0.866 |
| RE6 |  |  |  | 0.835 |
| RE7 |  |  |  | 0.860 |

The findings of Cronbach alpha, composite reliability, and average variance extracted are determined in this research. These findings are used to test the validity and reliability of research data significantly. The average variance extracted findings are used to test the variable between research data, and the findings loaded more than 0.50 are significantly accepted for it (Alarcón, Sánchez, & De Olavide, 2015). Furthermore, the findings of composite reliability are used to test the reliability of research data, and the findings of more than 0.70 are accepted as significant (dos Santos & Cirillo, 2021). Finally, the findings of Cronbach alpha are used to test the validity of the research data, and the findings loaded more than 0.70 are significantly accepted (Tavakol & Dennick, 2011). In this way, the data reported in Table 3 highlighted that the significant values for all three thresholds of the factors are achieved, and the data collected for this research has reliability and validity. These findings are also shown in Figure 3.

Cronbach Alpha, Composite Reliability and Average Variance Extracted

1.000

0.900

0.800

0.700

0.600

0.500

0.400

0.300

0.200

0.100

0.000

CSR

Industry Type

Leadership Style

Regulatory Environment

Cronbach's Alpha

Composite Reliability

Average Variance Extracted

Figure 3. Cronbach Alpha, Composite Reliability, and Average Variance Extracted

Table 3. Cronbach Alpha, Composite Reliability, and Average Variance Extracted

| Variables | Cronbach's Alpha | Composite  Reliability | Average Variance  Extracted |
| --- | --- | --- | --- |
| CSR | 0.840 | 0.892 | 0.638 |
| Industry Type | 0.875 | 0.904 | 0.574 |
| Leadership Style | 0.908 | 0.929 | 0.688 |
| Regulatory Environment | 0.903 | 0.924 | 0.638 |

The discriminant validity of this research data is also tested because it is necessary to confirm that the data for this research is appropriate. The findings of discriminant validity are tested with Heteritrait-Monotrait (HTMT) method. This method is based on a second generation of data analysis. The findings in HTMT analysis should not be more than 0.90 for significant discriminant validity (Gold, Malhotra, & Segars, 2001). The analyzed data showed that the findings of this research have appropriate discriminant validity as the data for this research has achieved significant HTMT. The results of HTMT are shown in Figure 4 and Table 4.

HTMT

2.5

2

1.5

1

0.5

0

CSR

Industry Type

Leadership Style

Regulatory Environment

CSR	Industry Type	Leadership Style	Regulatory Environment

Figure 4. HTMT

Table 4. HTMT

| Variables | CSR | Industry Type | Leadership  Style | Regulatory  Environment |
| --- | --- | --- | --- | --- |
| CSR |  |  |  |  |
| Industry Type | 0.762 |  |  |  |
| Leadership Style | 0.643 | 0.758 |  |  |
| Regulatory Environment | 0.665 | 0.776 | 0.773 |  |

The approach of cross-loadings is also tested to determine the findings of discriminant validity. These findings are significantly used to determine the discrimination between the data of each

measurement item. Simply, this method is used to test the discriminant validity at the measurement items level. However, the factor of cross-loadings is significant when the findings of one variable’s measurement items are more than the findings of other variables’ measurement items that are correlated with it (Barlat et al., 2013). The results of cross-loadings highlighted that this research has achieved significant discriminant validity and data is shown in Table 5. The findings of cross- loadings are also displayed in Figure 5.

Cross Loadings

1

0.9

0.8

0.7

0.6

0.5

0.4

0.3

0.2

0.1

0

CSR	Industry Type	Leadership Style	Regulatory Environment

Figure 5. Cross Loadings

CSR1 CSR2 CSR3 CSR4 CSR5 IT1 IT2 IT3 IT4 IT5 IT6 IT7 LS1 LS2 LS3 LS4 LS5 LS6 RE1 RE2 RE3 RE4 RE5 RE6 RE7

Table 5. Cross Loadings

| Items | CSR | Industry Type | Leadership Style | Regulatory  Environment |
| --- | --- | --- | --- | --- |
| CSR1 | **0.904** | 0.603 | 0.511 | 0.763 |
| CSR2 | **0.881** | 0.589 | 0.513 | 0.757 |
| CSR3 | **0.916** | 0.583 | 0.507 | 0.782 |
| CSR4 | **0.788** | 0.450 | 0.483 | 0.692 |
| CSR5 | **0.374** | 0.329 | 0.234 | 0.300 |
| IT1 | 0.348 | **0.637** | 0.551 | 0.435 |
| IT2 | 0.461 | **0.684** | 0.417 | 0.444 |
| IT3 | 0.501 | **0.802** | 0.584 | 0.571 |
| IT4 | 0.492 | **0.821** | 0.622 | 0.581 |
| IT5 | 0.528 | **0.778** | 0.453 | 0.552 |
| IT6 | 0.492 | **0.730** | 0.439 | 0.475 |
| IT7 | 0.598 | **0.831** | 0.532 | 0.585 |
| LS1 | 0.254 | 0.451 | **0.656** | 0.355 |
| LS2 | 0.425 | 0.542 | **0.875** | 0.562 |
| LS3 | 0.490 | 0.566 | **0.849** | 0.568 |
| LS4 | 0.499 | 0.532 | **0.874** | 0.608 |
| LS5 | 0.563 | 0.646 | **0.853** | 0.680 |
| LS6 | 0.563 | 0.618 | **0.849** | 0.672 |

| RE1 | 0.426 | 0.530 | 0.573 | **0.630** |
| --- | --- | --- | --- | --- |
| RE2 | 0.607 | 0.637 | 0.729 | **0.731** |
| RE3 | 0.691 | 0.561 | 0.489 | **0.806** |
| RE4 | 0.730 | 0.477 | 0.445 | **0.836** |
| RE5 | 0.732 | 0.554 | 0.543 | **0.866** |
| RE6 | 0.749 | 0.516 | 0.485 | **0.835** |
| RE7 | 0.804 | 0.580 | 0.517 | **0.860** |

The outcomes of the structural model are used for hypotheses testing. The findings of t-statistics are used to determine the rejection and acceptance of relationships. The findings of hypothesis 1 reported that industry type has a positive and significant impact on the regulatory environment. Furthermore, the findings of this research reported that hypothesis 2 is accepted and leadership style has a positive and significant impact on the regulatory environment. Thirdly, the findings of hypothesis 3 reported that the regulatory environment has a positive and significant impact on corporate social responsibility. Fourthly, the findings of hypothesis 4 highlighted that the regulatory environment has positive and significant mediation between industry type and corporate social responsibility. Lastly, the outcomes of hypothesis 5 demonstrated that the regulatory environment has a positive and significant mediation between leadership style and corporate social responsibility. Hence, all five theoretically developed relationships of this research are significantly accepted. The findings are presented in Table 6. However, the graphical representation of these findings is available in Figure 6.

Hypotheses Testing

50

40

30

20

10

0

Original Sample

Standard Deviation

T Statistics

P Values

Industry Type -> Regulatory Environment Leadership Style -> Regulatory Environment Regulatory Environment -> CSR

Industry Type -> Regulatory Environment -> CSR

Leadership Style -> Regulatory Environment -> CSR

Figure 6. Hypotheses Testing

Table 6. Hypotheses Testing

| No | Hypotheses | Original  Sample | Standard  Deviation | T  Statistics | P Values |
| --- | --- | --- | --- | --- | --- |
| 1 | Industry Type -> Regulatory  Environment | 0.391 | 0.065 | 5.995 | 0.000 |

| 2 | Leadership Style -> Regulatory  Environment | 0.443 | 0.063 | 7.057 | 0.000 |
| --- | --- | --- | --- | --- | --- |
| 3 | Regulatory Environment -> CSR | 0.856 | 0.020 | 42.617 | 0.000 |
| 4 | Industry Type -> Regulatory  Environment -> CSR | 0.334 | 0.058 | 5.791 | 0.000 |
| 5 | Leadership Style -> Regulatory  Environment -> CSR | 0.379 | 0.055 | 6.894 | 0.000 |

Significant: t > 1.96

# 5. Discussion and Conclusion

The findings of this research are taken with the data collected with the structural equation model. The hypotheses testing is done with t-statistics to determine the findings of this research. The empirical data for this research is considered appropriate to support the theoretically developed relationships. This research highlighted that industry type has a significant and positive impact on the regulatory environment. However, this relationship is new in the knowledge but has some theoretical support from the findings of existing studies in the literature. According to Sadiq et al. (2022), the preservation of the environment is a duty shared by all sectors of society. The sustainability of the environment is essential when any industry strives to advance its methods. In the current day, environmental control is essential for the sustainability of the environment. According to Dongfang et al. (2022), when the management of any organization is committed to working towards it, environmental sustainability can succeed. Additionally, the government's environmental safety regulations are appropriate to ensure the operation of organizations that promote environmental advancement. According to Hao et al. (2020), for improved advantage and more advanced operation, suitable activities for environmental sustainability must be taken over time. Once the leadership of an enterprise is committed to enhancing its sustainability, its environmental concerns can be trusted. According to Poursoleyman et al. (2023), to attain the objectives of ecological responsibility, modern industries must operate in the most environmentally friendly approaches. The CSR objectives are also a good method to promote environmental sustainability. To move in the direction of environmental sustainability, organizational performance is essential. According to Sun and Li (2021), any government's environmental protection regulations must be put into practice. Government laws' dependability for measuring organizational performance has grown over time.

Secondly, this research highlighted that leadership style has a significant and positive impact on the regulatory environment. Nevertheless, this relationship is new in the knowledge but has some theoretical support from the findings of existing studies in the literature. According to Xu et al. (2023), organizational performance must be used as a management tool to oversee the application of governmental rules for the accomplishment of sustainable goals. According to Waheed et al. (2022), any industry must have a proactive stance in support of environmental sustainability. Many organizations are making great strides in improving their organizational sustainability practices. According to Huang et al. (2020), the key to improving the efficiency of organizations is for the administration to be aware of the importance of achieving organizational goals. The best method to move forward in achieving organizational objectives for environmental sustainability is through steady organizational operations. According to Hu et al. (2022), government rules that are used to enhance organizational working can be improved upon to provide more effective tactics for enhancing organizational performance. The more effective means of enhancing organizational performance may contribute to greater environmental sustainability. According to D. Zhang et al.

(2022), the performance of these organizations is very important in the improvement of the environment, and many businesses are working in the right direction to fulfill their organizational goals of sustainably. The key to improving the efficiency of organizations is for the administration to be aware of the importance of achieving organizational goals. According to Teng et al. (2021), the best method to move forward in achieving organizational objectives for environmental sustainability is through steady organizational operations. Government rules that are used to enhance organizational working can be improved upon to provide more effective tactics for enhancing organizational performance.

Thirdly, this research highlighted that the regulatory environment has a significant and positive impact on CSR. Though, this relationship is new in the knowledge but has some theoretical support from the findings of existing studies in the literature. According to Liu et al. (2020), the more effective means of enhancing organizational performance may contribute to greater environmental sustainability. According to H. Li et al. (2022), the performance of these organizations is very important in the improvement of the environment, and many businesses are working in the right direction to fulfill their organizational goals of sustainably. The rate of development of organizational performance can help to advance ecological sustainability. According to Wei et al. (2022), any industry can better achieve environmental goals by improving employee behavior through environmental knowledge. When any organization works to attain environmental sustainability, industrial aims are also achieved. According to Abbas et al. (2023), by developing practical measures to boost organizational performance, modern businesses are primarily concerned with addressing environmental challenges. The leadership position is essential to improving organizational performance. Accordingly, the way forward for achieving the environmental goals is for staff members and managers to be aware of them. The path forward to achieving sustainability in it is the dependability of environmental concerns and knowledge of management for organizational goals.

The fourth hypothesis findings highlighted that the mediating role of the regulatory environment has a significant influence on the relationship between industry type and CSR. This relationship is at the infancy stage, but the empirical findings supported this relationship. The findings of existing studies in the literature also supported these relationships in the literature. According to Hsiao, Jiang, et al. (2022), management effectiveness and organizational practices are enhanced to meet these objectives when the organization's goals are to advance the environment. The organizational objectives have a direct impact on the success rate of industrial performance for the implementation of CSR. According to L. Zhong et al. (2022), the understanding of organizational sustainability is attained when the aims of any organization are moving in the proper path. The achievement of organizational goals depends on management performance improvement. According to Jaiyeoba et al. (2023), a better approach to achieving organizational sustainability may be to increase employee environmental awareness through organizational efforts. Personnel can be inspired to work in the appropriate direction for the accomplishment of sustainability goals by improvements in organizational goals and the value of organizational work for environmental protection. According to Hsiao, Huang, et al. (2022), a lot of organizations are making fair efforts to meet sustainability objectives, and these organizations need to safeguard the surroundings. The best path forward for organizational growth is through the steady operation of top management and long-term plans for organizational sustainability.

Finally, the fifth hypothesis findings highlighted that the mediating role of the regulatory environment has a significant influence on the relationship between leadership style and CSR. At

the same time, this relationship is in the infancy stage, but the empirical findings supported this relationship. The findings of existing studies in the literature also supported these relationships in the literature. According to Freze et al. (2023), any industry's management and employees putting in consistent work could be a step in the right direction for achieving CSR objectives. Each industry's leadership working style has the potential to both positively and negatively impact organizational performance. According to M. Zhong et al. (2022), a lot of executives influence their followers to inspire them to perform better for the organizations. Any organization that wants to attain organizational sustainability and environmental growth must engage in CSR. When an organization's goals are achievable and sustainable, environmental concerns are addressed. According to Hao et al. (2020), to improve organizational performance for environmental improvement, a better working methodology is required. Every industry has a responsibility to work on the regulations required for organizational sustainability. According to Broadstock et al. (2021), organizations that have created adequate policies for achieving sustainability are expected to operate more effectively towards achieving organizational objectives. Over time, the dependability of organizational performance either increases or decreases. According to B. Li et al. (2021), any company can have a safe working environment if its employees are highly motivated to do so. A better method to attain environmental advancement is through the consistency of organizational functioning and its advanced effort for organizational aims. According to Y. Zhang et al. (2023), to improve the direction of organizational goals, organizational management should design appropriate policies for the safety of the environment and implement these policies.

# 6. Theoretical and Practical Implications

The findings of this research are important from theoretical as well as practical perspectives. This research has extended the knowledge in the light of stakeholder theory. This research has introduced newly develop relationships in the literature. Firstly, this study has introduced in the literature that industry type has a positive impact on the regulatory environment, and this relationship was not reported in the literature before this research. Furthermore, this study added to the literature that the leadership style has a positive impact on the regulatory environment. In the same way, this relationship is newly introduced in the literature, and not reported by the findings of existing studies. Thirdly, this research demonstrated in the literature that the regulatory environment has a positive impact on corporate social responsibility. Meanwhile, this relationship is also new in the literature and not reported by the existing studies. This research furthermore highlighted that the regulatory environment has positive mediation between industry type and corporate social responsibility. Finally, the study highlighted in the literature that the regulatory environment has positive mediation between leadership style and corporate social responsibility. In this way, the relationships developed by this research are worthy of knowledge because new directions regarding corporate social responsibility under the light of stakeholder theory are highlighted by this research.

The findings of this research have practical implications as the findings demonstrated that environmental regulations are possible with industry type and leadership style. In this way, the working of leadership and their style matters a lot regarding environmental issues. The success and failure of environmental regulations are based on the working practices of the leadership. These regulations are required to be improved over time for achieving corporate social responsibility. Firstly, the primary industry is required to work on the environmental improvement that is necessary to achieve sustainability in it. The primary industries are required to improve the

efficiency in the utilization of natural resources which is a way forward to get success in it. In the meanwhile, the secondary industry is also required to work to advance the efficiency in the utilization of natural resources with the external factors that are critical to managing for better work. On the other hand, the industry of the tertiary sector is also required to be improved for the utilization of resources to protect the environment. In the meanwhile, access to natural resources protection for the safety of the environment is required to be improved by every type of industry. In this way, corporate social responsibility can be achieved significantly to get better output.

# 7. Future Directions

Based on the empirical results, this study highlighted that the impact of industry type and leadership style is significant on the regulatory environment and CSR. The study also introduced the regulatory environment as a mediator between leadership style, industry type, and CSR. Moreover, the framework of this research is based on the research gap, and the novel findings of this study are significant addiction to the knowledge. The study has presented remarkable theoretical implications in the body of knowledge. On the other hand, the research also asserted practical implications that are reliable to advance the practices for the regulatory environment and achieving CSR by Chinese industries. This study has several limitations that are required to be addressed significantly for future directions to improve the body of knowledge. This study has limitations in its data collection because the data was collected only from the management of the industries in China. However, they understand the relationship between leadership style, industry type, and regulatory environment, so the data collected from the employees would be appropriate for the findings. In this way, this limitation of research must be addressed by future studies to contribute significant findings to the literature. In the meanwhile, this research has not considered environmental awareness as a factor in the research framework. This exclusion of environmental awareness limits the findings of this research. In this way, future studies are required to consider environmental concern as moderating variable to determine the findings and contribute to the literature. This research has collected cross-sectional data for its findings, but future studies are required to collect longitudinal data for the findings in research. Thus, the findings of this research would be compared with the findings of other research that used to collect data longitudinally. Also, the respondents from other regions such as Russia can be targeted for data collection for the significant findings in the body of knowledge.

# Statements and Declarations

**Author Contributions**

Conceptualization, YZ, and SK; Investigation, YZ & SK; Methodology, SK & MS; Project administration, YZ; Supervision & Validation, YZ; Writing original draft, SK, YZ & MS; Writing

– review & editing, SK & MS.

All authors have read and agreed to the published version of the manuscript.

# Funding

No specific funding was received for publication of this article

# Institutional Review Board Statement

This study adheres to the guidelines of the ethical review process of the associated university and approved by the ethics committees of the School of Management Science and Engineering, Zhengzhou University, Henan, China.

# Informed Consent Statement:

Verbal Informed consent was taken before data collection.

# Competing Interests

There is no competing interest to disclose

# Availability of data and materials

Data associated with this study is included in the article. However, further inquiries can be directed to the corresponding author upon a reasonable request.

# Conflicts of Interest

The authors declare no conflict of interest

# Acknowledgments

We acknowledge the efforts of editor and reviewers for their valuable comments and feedback to improve the quality of paper.

# References

Abbas, J., Wang, L., Belgacem, S. B., Pawar, P. S., Najam, H., & Abbas, J. (2023). Investment in renewable energy and electricity output: Role of green finance, environmental tax, and geopolitical risk: Empirical evidence from China. *Energy, 269*, 126683.

Alarcón, D., Sánchez, J. A., & De Olavide, U. (2015). *Assessing convergent and discriminant validity in the ADHD-R IV rating scale: User-written commands for Average Variance Extracted (AVE), Composite Reliability (CR), and Heterotrait-Monotrait ratio of correlations (HTMT).* Paper presented at the Spanish STATA meeting.

Barlat, F., Ha, J., Grácio, J. J., Lee, M.-G., Rauch, E. F., & Vincze, G. (2013). Extension of homogeneous anisotropic hardening model to cross-loading with latent effects. *International Journal of Plasticity, 46*, 130-142.

Broadstock, D. C., Chan, K., Cheng, L. T., & Wang, X. (2021). The role of ESG performance during times of financial crisis: Evidence from COVID-19 in China. *Finance Research Letters, 38*, 101716.

Dongfang, W., Ponce, P., Yu, Z., Ponce, K., & Tanveer, M. (2022). The future of industry 4.0 and the circular economy in Chinese supply chain: In the Era of post-COVID-19 pandemic. *Operations Management Research, 15*(1-2), 342-356.

dos Santos, P. M., & Cirillo, M. Â. (2021). Construction of the average variance extracted index for construct validation in structural equation models with adaptive regressions. *Communications in Statistics- Simulation and Computation*, 1-13.

Freeman, R. E., Harrison, J. S., Wicks, A. C., Parmar, B. L., & De Colle, S. (2010). Stakeholder theory: The state of the art.

Freze, T., Korneev, A., Krayneva, R., Oruch, T., Kandalov, W., & Strielkowski, W. (2023). Business Leadership and Corporate Social Responsibility in the Post-COVID Era. *Economies, 11*(3), 98.

Friedman, A. L., & Miles, S. (2006). *Stakeholders: Theory and practice*: OUP oxford.

Gold, A. H., Malhotra, A., & Segars, A. H. (2001). Knowledge management: An organizational capabilities perspective. *Journal of management information systems, 18*(1), 185-214.

Hao, F., Xiao, Q., & Chon, K. (2020). COVID-19 and China’s hotel industry: Impacts, a disaster management framework, and post-pandemic agenda. *International Journal of Hospitality Management, 90*, 102636.

Hsiao, C.-Y., Huang, S.-M., Kang, Y.-Y., & Li, Y.-X. (2022). The Impact of ESG Performance on Business Performance before and after the COVID-19-Taking the Chinese Listed Companies as a Sample. *Asian Journal of Economics, Business and Accounting, 22*(5), 56-68.

Hsiao, C.-Y., Jiang, Q., Lian, L.-Y., & Wang, Y.-S. (2022). The Impact of Environment, Social, and Governance (ESG) Performance on the Change of Z-score before and after the COVID-19–the Case of Chinese A-Share Manufacturing Industry Companies. *Asian Journal of Economics, Business and Accounting, 22*(6), 46-57.

Hu, Y., Qu, S., Huang, K., Xue, B., & Yu, Y. (2022). The Chinese plug-in electric vehicles industry in post- COVID-19 era towards 2035: Where is the path to revival? *Journal of Cleaner Production, 361*, 132291.

Hua, L., Rasool, Z., Ansari, M. A. A., Khan, A. J., Hanif, N., & Ul Hameed, W. (2023). International Students’ Nostalgic Behaviour towards the Purchase of Products and Services. *Sustainability, 15*(4), 2888.

Huang, W., Chen, S., & Nguyen, L. T. (2020). Corporate social responsibility and organizational resilience to COVID-19 crisis: An empirical study of Chinese firms. *Sustainability, 12*(21), 8970.

Jaiyeoba, H. B., Hossain, S., Mohd Salleh, H., & Elgharbawy, A. A. (2023). Corporate social responsibility as an effective promotional tool for the Malaysian halal certified companies in the era of Covid-19. *Journal of Islamic Marketing, 14*(2), 317-341.

Li, B., Zhong, Y., Zhang, T., & Hua, N. (2021). Transcending the COVID-19 crisis: Business resilience and innovation of the restaurant industry in China. *Journal of Hospitality and Tourism Management, 49*, 44-53.

Li, H., Hu, Q., Zhao, G., & Li, B. (2022). The co-evolution of knowledge management and business model transformation in the post-COVID-19 era: insights based on Chinese e-commerce companies. *Journal of Knowledge Management, 26*(5), 1113-1123.

Liu, J., Qiao, P., Ding, J., Hankinson, L., Harriman, E. H., Schiller, E. M., . . . Zhang, H. (2020). Will the aviation industry have a bright future after the COVID-19 outbreak? Evidence from Chinese airport shipping sector. *Journal of Risk and Financial Management, 13*(11), 276.

Poursoleyman, E., Mansourfar, G., Hassan, M. K., & Homayoun, S. (2023). Did corporate social responsibility vaccinate corporations against COVID-19? *Journal of Business Ethics*, 1-27.

Royston, P. (1992). Which measures of skewness and kurtosis are best? *Statistics in Medicine, 11*(3), 333- 343.

Sadiq, M., Nonthapot, S., Mohamad, S., Chee Keong, O., Ehsanullah, S., & Iqbal, N. (2022). Does green finance matter for sustainable entrepreneurship and environmental corporate social responsibility during COVID-19? *China Finance Review International, 12*(2), 317-333.

Shevlin, M., & Miles, J. N. (1998). Effects of sample size, model specification and factor loadings on the GFI in confirmatory factor analysis. *Personality and Individual Differences, 25*(1), 85-90.

Sun, Y., & Li, Y. (2021). COVID-19 Outbreak and financial performance of Chinese listed firms: Evidence from corporate culture and corporate social responsibility. *Frontiers in Public Health, 9*, 710743.

Taherdoost, H. (2016). Sampling methods in research methodology; how to choose a sampling technique for research. *How to Choose a Sampling Technique for Research (April 10, 2016)*.

Tang, H., Maqbool, A., Khan, A. J., Ul Hameed, W., Batool, H., & Ahmed, T. (2023). The moderating role of sustainable development goals in reviving the economy through green initiatives. *Frontiers in Environmental Science, 11*, 509.

Tavakol, M., & Dennick, R. (2011). Making sense of Cronbach's alpha. *International journal of medical education, 2*, 53.

Teng, X., Teng, Y.-M., Wu, K.-S., & Chang, B.-G. (2021). Corporate social responsibility in public health during the COVID-19 pandemic: quarantine hotel in China. *Frontiers in Public Health, 9*, 620930.

Waheed, A., Shehzad, F., Arif, F., Abbas, A. A., Mehmood, Z., & Usman, M. (2022). Role of CSR in achieving customers’ satisfaction and retention during COVID-19 and post-pandemic period: Empirical evidence from emerging nations. *Human Systems Management*(Preprint), 1-13.

Wei, R., Ayub, B., & Dagar, V. (2022). Environmental benefits from carbon tax in the Chinese carbon market: a roadmap to energy efficiency in the post-COVID-19 era. *Frontiers in Energy Research, 10*.

Xu, N., Chen, J., Zhou, F., Dong, Q., & He, Z. (2023). Corporate ESG and resilience of stock prices in the context of the COVID-19 pandemic in China. *Pacific-Basin Finance Journal*, 102040.

Zhang, D., Lu, S., Morse, S., & Liu, L. (2022). The impact of COVID-19 on business perspectives of sustainable development and corporate social responsibility in China. *Environment, Development and Sustainability*, 1-24.

Zhang, Y., Li, J., Lei, Y., & Yan, T. (2023). Explore the approaches to corporate social responsibility implemented by e-commerce platforms in china during the early stage of covid-19: a mixed- methods content analysis. *Disaster Medicine and Public Health Preparedness, 17*, e76.

Zhong, L., Sun, S., Law, R., Li, X., & Yang, L. (2022). Perception, reaction, and future development of the influence of COVID-19 on the hospitality and tourism industry in China. *International Journal of Environmental Research and Public Health, 19*(2), 991.

Zhong, M., Zhao, W., & Shahab, Y. (2022). The philanthropic response of substantive and symbolic corporate social responsibility strategies to COVID-19 crisis: Evidence from China. *Corporate Social Responsibility and Environmental Management, 29*(2), 339-355.
