# Supplementary material for: From crisis to responsibility: The role of industry type, leadership style, and regulatory environment in shaping post-COVID-19 CSR initiative
Source: PLoS One. 2024 Apr 18;19(4):e0292732. doi: 10.1371/journal.pone.0292732 (PMC11025823; doi:10.1371/journal.pone.0292732)
Supplement: Attachment — Submitted filename: Response Sheet plos one.docx [file pone.0292732.s003.docx]

Dear Editor and Reviewers,

Reference to the Manuscript PONE-D-23-20140 titled **“From Crisis to Responsibility: The Role of Industry Type, Leadership Style, and Regulatory Environment in Shaping Post-COVID-19 CSR Initiative”**. The reviewer 1 and review 2 both recommended the publication of this paper with minor revisions. As advised by the worthy referees of Plos one, suggested changes have been incorporated into the manuscript.

Note: The Red highlighted text indicates the revisions made in the manuscript. We also thoroughly checked and proof read the article and the proof-reading changes are indicated with the blue color.

We hope, the revised manuscript will satisfy the journal’s requirements for publication.

Thanks, and Best Regards,

The Authors

**Reviewer #1:**

**Comment:**

What novelties are offered?
**Response:**

The novelty of research is explained in the introduction section of the article. This research offered novelty based on its findings as it reported CSR initiatives in post-pandemic environment are possible with the type of industry and the style of leadership. These both factors are significant for CSR practices, the regulatory environmental factors are supporting leadership style to lead the work to advance CSR practices.

**Comment:**

An abstract summarizes, usually in one paragraph of 200-259 words or less, the major aspects of the entire paper in a prescribed sequence that includes: 1) the overall purpose of the study and the research problem(s) you investigated; 2) the basic design of the study; 3) major findings or trends found as a result of your analysis; and, 4) a brief summary of your interpretations and conclusions.
**Response:**

Dear reviewer, the abstract of the paper is already written according to the standard guidelines. Thanks for your valuable comments.

**Comment:**

Authors must add citation to these sentences from this article: https://journal.ppishk.org/index.php/jcgpp/article/view/183
**Response:**

Respected reviewer, the said paper is not fit in the scope of the current scientific research. Therefore, we extend our apology to add this reference. If you have further concerns, please inform us.

**Comment:**

Please add state of the art, gap analysis, and purpose of the paper.

**Response:**

Respected reviewer, thanks for your comments. The state-of-the-art gap and purpose of the study is further explained in the introduction section of the study. Please find the highlighted changes.
**Comment:**

Methodology is Clear!
**Response:**

Dear reviewer, thanks for your understanding and appreciating our work

**Comment:**

Please add more references from journal articles!
**Response:**

Respected reviewer, the references are updated as per your recommendations. Thanks for your valuable feedback.

**Comment:**

Please update your references!

**Response:**

Dear reviewer, thanks for your comments. All references are cited by using end note software. All reference are checked and verified as per the recommendations of worthy reviewer.

**Reviewer #2:**

**Comment:**

Minor adjustments are suggested in the conclusion section, with the need to be firm in distinguishing closing points. The ending should be totally revised with the following points in mind:
Brief description of the research topic, copyright gained during the investigation, generalisation of research findings (where each point should be devoted to as an answer provided in the Introduction as mentioned in the Introduction)

**Response:**

Respected reviewer, thanks for your valuable comments. We are thankful for your critical commentary on this scientific research. In this way, the conclusion section is revised as per your recommendations. Highlighted changes are indicated with red highlighted text.
